# Recent Prospects of Carbonaceous Nanomaterials-Based Laccase Biosensor for Electrochemical Detection of Phenolic Compounds

**DOI:** 10.3390/bios13030305

**Published:** 2023-02-22

**Authors:** Sakshi Verma, Deeksha Thakur, Chandra Mouli Pandey, Devendra Kumar

**Affiliations:** 1 Department of Applied Chemistry, Delhi Technological University, Delhi 110042, India; 2 Department of Chemistry, Faculty of Science, SGT University, Gurugram 122505, India

**Keywords:** laccase, biosensor, electrochemical, carbonaceous material, phenolic compounds

## Abstract

Phenolic compounds (PhCs) are ubiquitously distributed phytochemicals found in many plants, body fluids, food items, medicines, pesticides, dyes, etc. Many PhCs are priority pollutants that are highly toxic, teratogenic, and carcinogenic. Some of these are present in body fluids and affect metabolism, while others possess numerous bioactive properties such as retaining antioxidant and antimicrobial activity in plants and food products. Therefore, there is an urgency for developing an effective, rapid, sensitive, and reliable tool for the analysis of these PhCs to address their environmental and health concern. In this context, carbonaceous nanomaterials have emerged as a promising material for the fabrication of electrochemical biosensors as they provide remarkable characteristics such as lightweight, high surface: volume, excellent conductivity, extraordinary tensile strength, and biocompatibility. This review outlines the current status of the applications of carbonaceous nanomaterials (CNTs, graphene, etc.) based enzymatic electrochemical biosensors for the detection of PhCs. Efforts have also been made to discuss the mechanism of action of the laccase enzyme for the detection of PhCs. The limitations, advanced emerging carbon-based material, current state of artificial intelligence in PhCs detection, and future scopes have also been summarized.

## 1. Introduction

Phenolic compounds (PhCs) are extensively used in the production of dyes, chemical analysis, disinfectants, synthesis of artificial resins, petrochemicals, pharmaceuticals, textiles, paints, and medical industries [1,2]. These PhCs are present in body fluids, and certain PhCs have potential antioxidant properties [3]. A diverse variety of PhCs display varied impacts on biotic and abiotic components.

PhCs used for industrial purposes and a few released from the body (e.g., estrogen) are ubiquitous pollutants that are toxic even at low concentrations. The level of PhCs toxicity is in the range of 9–25 mg L^−1^, while the water purity standards set by the US EPA allow a threshold value of approximately 1 ppb of phenol in the case of surface water [1]. The European Commission has prescribed a 0.001 mg L^−1^ concentration limit of PhCs in natural water, whereas the Central Pollution Control Board of India has restricted 1.0 mg L^−1^ of a phenolic compound as the benchmark for discharge of water to inland surfaces under the Environment (Protection) Rules, 1986. When discharged into the environment without prior treatment, these compounds lead to severe complications and long-term health issues for humans, animals, and marine systems [4]. Drinking water containing PhCs may cause diseases such as indigestion, skin burns, muscle tremor, liver problems and kidney damage in humans, and death of fish in aquatic regions [2]. In contrast, some other PhCs present in food and body fluid are responsible for preventing disease by balancing blood pressure and stress, reducing cardiovascular diseases, and providing healthy antioxidants [5,6]. The effects and sources of widely used PhCs have been summarized in Table 1.

**Figure 1:**
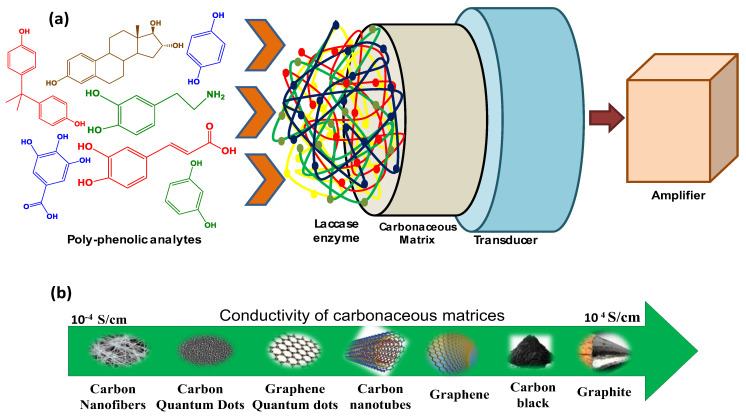
Scheme representing (**a**) a biosensor for PhCs detection using a carbon-based matrix to support the laccase enzyme and (**b**) the comparative conductivity of carbon−based materials.

Various chromatographic and spectroscopic techniques have been used to detect and quantify PhCs. These laboratory techniques require tedious process of sample pretreatment from water bodies, which are time-consuming and costly [7]. For rapid and efficient detection, it is necessary to develop portable and more sensitive on-site tools comprising resourceful materials to reduce the devastating effects caused by PhCs. In this context, biosensors can be considered the most reliable techniques for on-site and specific detection of PhCs [8] (Figure 1a). 

**Table 1:** Harmful effects and sources of widely used PhCs on humans and animals.

S.No.	PhCs	Effects	Sources	Ref.
1.	Catechol	Protein destruction in the body Damage to DNA	Coal conversion process Crude wood tar Coal tar production	[9]
2.	Bisphenol A	Endocrine troublesome effects Interruption of the onset of puberty	Food and beverage packaging Flame retardants Building materials and electronic components Paper coatings	[10]
3.	Caffeic and dihydrocaffeic acids	Damages DNA in the presence of copper Antioxidant property Prevents cardiovascular disease Reduces stress	Wine Coffee Spices and herbs	[11]
4.	Chlorophenol	Mouth scorching Necrotic gashes in the respiratory canal	Bleaching Iron and steel industries Paper pulp and paper board mills Dye and pharmaceutical industries	[12]
5.	Hydroquinone	Damages chromosome Toxic to soil microbial activities Skin irritation	Food and Rubber industries as an antioxidant Paint and fuel industries as a stabilizer Cosmetic industries	[13]
6.	Para-cresol	Central nervous system disease Cardiovascular system Lungs and kidneys diseases	Fumigants Disinfectant Explosives	[14]
7.	Estrogen	Endocrine disability Breast cancer	Urine	[15]
8.	Dopamine	Enhance brain functions and memory Regulates blood pressure	Brain	[16]
9.	Galliac acid	Antioxidant Reduce the risk of cardiovascular diseases Prevent certain cancer	Wine Chestnut Berries	[17]

The advantages of using biosensors include high sensitivity, accuracy, short response time, reliability, longer shelf life, and user-friendliness [7,18,19,20]. Biosensors can detect a very low amount of contaminant even from a composite medium, (wastewater) and are considered reliable for quantifying the total phenolic content in food by checking antioxidant properties of the foodstuff [21,22]. Several bio-recognition components have been used to detect polyphenols, such as microorganisms [23], DNA [24], whole cells [18], anti-bodies, and enzymes [25]. For monitoring PhCs, enzymatic biosensors based on laccase (Lac) [26], horseradish peroxidase [27], and tyrosinase [28,29] have proven to be the most effective due to their ability to directly catalyze electron transfer reactions without introducing cofactors into the reaction medium. Different supporting matrices such as conducting polymers, metal and metal oxide nanoparticles, carbon nano tubes (CNTs), graphite, silica gel, activated charcoal, reduced graphene oxide (RGO), glass surfaces, etc., have been used for the immobilization of these enzymes [30]. The comparative conductivities of such materials have been shown in Figure 1b.

In the current review article, we discuss:

- The recent progress in carbon-based nanomaterials (CNMs)-based Lac biosensors;
- The structure, mechanism of action, and immobilization methods of the Lac enzyme on CNMs;
- The application of CNMs-based Lac biosensors for the detection of PhCs present in food and body fluids;
- The limitations of highly utilized graphitic materials and challenges.

Recently, few articles have focused on discussing the importance of Lac enzymes for many applications. However, to the best of our knowledge, none of them focus on the application of CNMs, which can resolve the major challenge in the development of Lac-based biosensors for the detection of PhCs.

## 2. Transduction Principle for Monitoring PhCs

The biosensor has emerged as an interesting analytical approach for the detection of PhCs. It mainly consists of two components, including biological entities (analyte and receptor) and a transducer [31]. Enzyme-based sensors are prominently explored due to their ease of selectivity, sensitivity, reliability, and usage [32,33]. In this context, Lac has been considered a greener bio-recognition component supported on modified conducting carbon-based matrices for the detection of PhCs. An enzymatic biosensor produces signals targeted to the analyte’s concentration by combining an enzyme with the transducer, and the transducer converts the signal received into a quantifiable response [34]. Amperometric biosensors aim to electrochemically convert non-active analytes into products by catalysis depending on the enzyme system that performs oxidation and reduction at the surface of the working electrode. Das et al. [35] fabricated Lac-based amperometric biosensor based on an osmium tetroxide/poly 4-vinyl pyridine/multi-walled carbon nanotube (MWCNT)/Nafion/carbon black/GCE electrode for pyrocatechol detection in environmental samples. Kavetskyy et.al. [36] investigated an electroconductive immobilized matrix based on microporous carbon fibers and the Lac enzyme using the amperometric transduction principle. A successive assessment of biosensors was performed by detecting industrial pollutant catechol from actual communal wastewater samples. There is a limited number of optical biosensors reported in the literature that use CNMs for the detection of PhCs, one of which comprises carbon quantum dots (C-QDs) and the Lac enzyme [37]. An optical biosensor based on a sol-gel immobilized Lac was also developed for the detection of three isomeric PhCs (catechol, resorcinol, and hydroquinone) in real and tap water samples [38].

### 2.1. Electron Transfer and Reaction Mechanism in Lac

**Figure 2:**
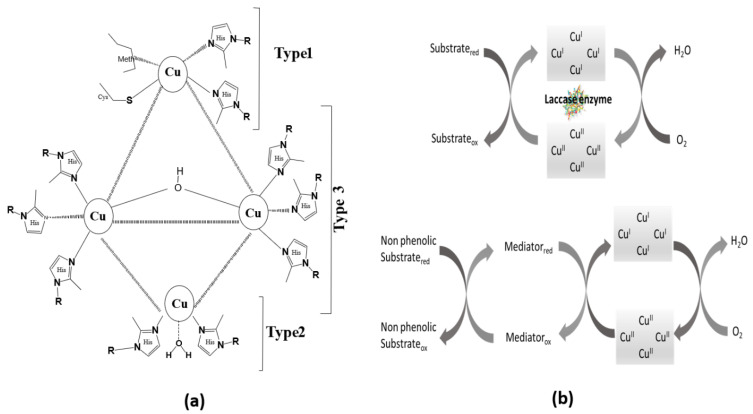
Scheme showing the (**a**) assembly of the Lac enzyme, adapted from ref. [39] and (**b**) the activity of the Lac enzyme for a phenolic and non-phenolic substrate [42].

Lac is the largest subgroup of multi-copper oxidases, comprising four copper atoms in different oxidation states, as shown in Figure 2a. Lac has the capability to catalytically oxidize a variety of PhCs in the presence of molecular oxygen by performing a four-electron reduction of oxygen to water. The substrate undergoes reduction by the Lac enzyme conjoining its four-electron oxidation leading to the reductive cleavage of molecular oxygen (O=O) bonds by four electrons along with four Cu atoms. The Cu atoms forming the core of Lac are sub-divided into three types, based on the electron paramagnetic resonance (EPR) spectroscopic technique in the literature [39,40]:

- Type 1 (Cu C1) has trigonal coordination with one sulfur atom of cysteine (cys) and two nitrogen atoms of histidine imidazole units. The fourth coordination is with S of methionine attached axially and far more than the other three. Therefore, the structure seems distorted in the form of tetrahedral geometry with triagonal elongation [41]. It possesses intense blue color resulting from the strong electronic absorbance at 600 nm (charge transfer: S of cysteine to Cu, ε = 5000 M^−1^ cm^−1^) in UV/visible spectroscopy, and its paramagnetic nature has been confirmed using EPR spectroscopy.
- Type 2 (Cu C2) has been found to be coordinated with two nitrogen atoms of histidine units and one water ligand. Although it is colorless, EPR studies have revealed its paramagnetic nature via ultrafine splitting.
- Type 3 (2Cu C3) comprises two anti-ferromagnetically coupled Cu atoms each tetragonally coordinated with three nitrogen atoms of histidine units and one bridged hydroxide group. The bridged hydroxide leads to electron-paired Cu sites. The oxidized form shows weak absorbance under UV-visible spectrum having a shoulder at around 330 nm (charge transfer: OH to Cu), and EPR studies show no signal signifying their diamagnetic nature.

The histidine units are, therefore, coordinated in a 2:2:6 ratio for Cu C1, Cu C2, and 2Cu C3, respectively. The bonds connecting Cu and N of histidine are slightly different in bond lengths. The approximate Cu–N bond length for all categories of Cu is 2 Å A֯, some Cu–N bonds are of greater bond length, and some are found to be slightly smaller. The distance between Cu C1 and binuclear Cu atoms (2Cu C3) is found to be around 12 Å, whereas, in the trinuclear Cu cluster, the distance between Cu C2 and binuclear Cu atoms (2Cu C3) is observed at about 4 Å.

The Electron Paramagnetic Resonance (EPR) of Lac have been observed to be perturbed in the presence of strong anion binding, leading to the disturbance of the EPR signal intensity of Cu C2 and in the anti-ferromagnetic coupling of 2Cu C3. At the same time, the electron reduction of molecular oxygen and discharge of water takes place at a tri-nuclear cluster of Cu C2 and 2Cu C3 atoms. Based on the physical and chemical properties of oxidoreductase Lac, the positioning of copper atoms has different well-defined redox potentials (E_0_). Depending upon the chemical nature, the Lac enzymes obtained from basidiomycetes (mostly white rot fungi) are considered to have high redox potential, whereas low redox potential is found to be possessed by Lac obtained from bacterial and plant sources [40]. Other biotic sources of Lac enzymes have been listed in Table S1. However, the physical structure shows that Cu C1 and 2Cu C3 have variable low (+E_0_ = 0.4–0.5 V) and high (E_0_ = 0.7–0.8 V) redox potentials with respect to standard hydrogen electrodes. The low and high redox potential of Cu C2 appears at approximately 0.4 V [43]. 

Biosensors based on tyrosinase, horseradish peroxidase, Lac, and polyphenol oxidase enzymes have been broadly used for the detection of different PhCs. However, Lac possesses higher stability and better catalytic ability for electron transfer reactions [44]. Lac-based biosensors are modest in assembling over other reported enzymatic biosensor because they do not necessarily involve hydrogen peroxide as one of the co-substrates or some other co-factors for the catalytic process. 

### 2.2. Activity of the Lac Enzyme

The catalytic activity of Lac depends on the copper (Cu) atoms. All the Cu sites show different functions. Cu C1 is the primary electron acceptor from the reduced substrate. The high redox potential (790 mV) of Cu C1 is the reason for substrate oxidation at its site [39]. Further, the electron received by Cu C1 is delivered via Cys-His pathways to the trinuclear Cu cluster that shows an inter-electron transfer mechanism, which takes place between Cu C1 and 2Cu C3 positions. Due to this transfer of an electron from one Cu site to another, the step where the oxygen molecule is reduced to water by four electrons replicates. It has been reported that H_2_O_2_ was not detected during the steady state [45]. At the same site, an oxidized form of the enzyme has been generated. During this aerobic oxidation process, 2Cu C3 accepts two electrons in the presence of Cu C2. The presence of Cu C2 is found to be necessary for the acceptance of electrons by 2Cu C3. In short, the Lac enzyme is oxidized by reducing oxygen to water followed by the oxidation of the substrate leading towards the terminus of the catalytic mechanism by reducing the oxidized Lac enzyme to Lac again. 

The Lac enzyme does not possess the ability to oxidize non-phenolic substrates directly. To increase the range of compounds that can be oxidized using the Lac enzyme, several Lac-based mediator compounds have been reported. These Lac-based mediator compounds consider the substrate as an intermediate, exhibiting higher redox potential and indirectly oxidizing non-phenolic substrates [43] (Figure 2b). The mediators enhance the catalytic performance of the Lac enzyme, acting as a co-catalyst. The catalytic mechanism for non-phenolic compounds initiates with the conversion of Lac into oxidized Lac on the same side, leading to the reduction of oxygen to release water. The mediator works by oxidizing the substrate and reducing itself to the original mediator, indicating completion of the co-catalytic mechanism [42]. A standard mediator must be low in molecular weight to avoid hindrance due to size and should possess a high redox potential to enhance the catalytic oxidation process. An efficient mediator must perform several consecutive cycles deprived of deterioration. For example, (2,2′-azino-bis(3-ethylbenzothiazoline-6-sulfonic acid)) is one of the most frequently used mediator for oxidation of non-phenolic compounds [43].

Enzymes undergo specific interactions with certain chemical carriers and substrates to undergo genetic and chemical modifications to increase their catalytic activity. In this context, Zhang et.al. reported that flower-like Cu_3_(PO_4_)_2_.3H_2_O nanocrystals integrated with Lac shows 6 times activation in the activity of the Lac enzyme [46]. Whereas, Wu et al. found a 1.5 to 4 times increase in the activity of fungal Lac after its pre-incubation with different organic solvents such as methanol, acetone, dimethylformamide, and dimethyl sulfoxide [47]. On the other hand, the influence on enzymatic activity has also been studied based on mutagenesis focusing on the residue at certain position and pH. Another method to enhance activity is through the use of multienzyme, which involves channeling different substrates, kinetics matching, and 3-D distribution of the involved enzymes. These methods can be utilized to engineer the best enzyme–substrate relationship to provide superior catalytic performance [46].

### 2.3. Immobilization Matrix

In addition to the several merits of the Lac enzyme, the use of the Lac enzyme for biosensor application has not been explored much because of the denaturation of the enzyme by the external environment. In order to increase the stability, lifetime, efficiency, and reusability, and to equilibrate the cost of developed biosensors for detection of PhCs, the efficient immobilization of the Lac enzyme onto suitable matrices is very important [48]. Immobilization provides a way to increase the ability of enzymes by reducing the cost of production [49], leading to better stability of the biosensor [50], strategies for reproducibility, ease of recovery [49], etc. Moreover, the involvement of new bonding, entrapment, and crosslinking also enhances the shelf life of enzymes [51,52]. Immobilization maintains the structural stability and functional possessions of the immobilized enzyme by proper attachment to the surface of the appropriate matrix so that the required activity of the enzyme can be retained throughout the repetitive use of sensor [53,54]. Immobilization methods vary from one enzyme to another and depend on the type of application for which the enzymes are being used. An ideal matrix must be physically rigid, chemically inert, and insoluble in enzymes to preserve its catalytic properties. The immobilization matrix should be thermally and photo-chemically stable, showing effective charge transfer capability. Furthermore, there must be permissible diffusion of a bio-catalytic reaction between the substrate and matrices on which the enzyme has to be immobilized [55]. 

**Figure 3:**
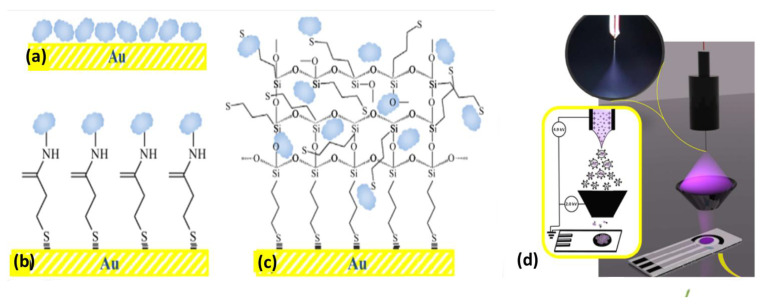
Enzyme immobilization methodologies employed for biosensor fabrication using Lac onto a Au matrix: (**a**) direct adsorption, (**b**) covalent binding, (**c**) sol-gel encapsulation, Adapted with permission from Ref. [56]. Copyright © 2013 Elsevier and (**d**) the electrospray deposition technique for Lac immobilization on carbon black-nanomodified SPE. Adapted with permission from Ref. [57]. Copyright © 2020 Elsevier.

Effective immobilization also helps to restrict the gross movement of biomolecules, thus leading to the fabrication of a stable and accurate biosensor. The different immobilization techniques include (i) covalent bonding, (ii) adsorption, (iii) cross-linking, (iv) entrapment, and (v) encapsulation (Figure 3a–c) [56]. One more method, i.e., the electrospray deposition technique, has also been reported for Lac immobilization on carbon black-nanomodified screen-printed electrodes, as shown in Figure 3d [57]. Several physical and chemical factors, such as pH, temperature, solubility, concentration, etc., influence the immobilization strategies. Large numbers of organic and inorganic matrices have been used for immobilizing the Lac enzyme. In particular, conducting polymers [58], metal nanoparticles [59], metal oxides [60], silica [61,62], clay [63], CNMs, metal–organic frameworks [64], etc. Conducting polymers (CPs) have been widely explored for the fabrication of Lac-based biosensors because of their good compatibility with enzymes and electron conduction ability. In the case of CPs, conductivity can also be varied by hosting doping materials. Calitri et al. [65] evaluated the total phenolic content using CPs poly(aniline-co-2-aminobenzylamine) supported on MWCNT by immobilizing Lac. Yaropolov et al. [66] fabricated a Lac biosensor using three CPs electrodes, viz., Nafion, poly(ethyleneimine), and Eastman AQ 29D supported with GCE for amperometric determination of PhCs. The synthesis of polydopamine nanoparticles based on a bacterial cellulose composite was also accomplished as a compatible matrix for Lac immobilization. Meso-porous silica sieve matrices possess compatibility and uniformity with respect to well-ordered pore structure, size, density, and surface characteristics. Amperometric detection of catechol has been reported where magnesium-doped mesoporous silica sieve–polyvinyl alcohol composite has been used as a matrix [67]. Li et al. constructed a disposable biosensor using silica sphere on the surface of MWCNT with a screen printed electrode (SPE) for immobilizing Lac and detecting dopamine [68]. The major problem with this sensor is the film formation ability and lower conductivity. 

**Figure 4:**
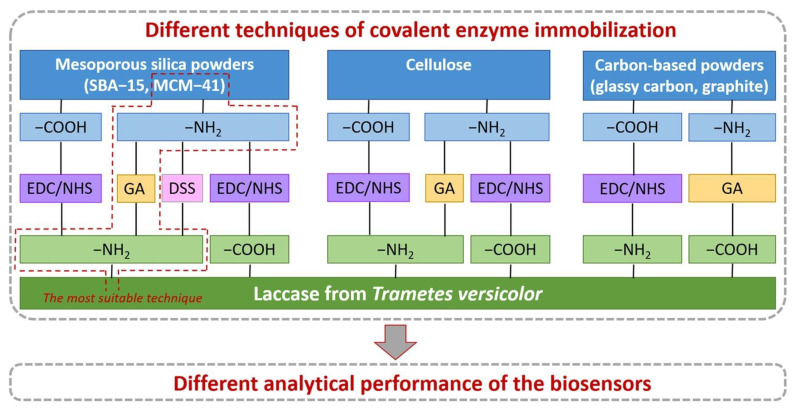
Illustration of different methods of covalent immobilization of laccase onto a variety of matrices Adapted with Permission from Ref. [70]. Copyright © 2022 Elsevier.

The recent era specifically focuses more on immobilization matrices based on nanomaterials. Although many other matrices have been used to support enzymes, nanomaterials-based matrices are widely explored because of their numerous benefits. Introducing nanomaterial matrices into the enzyme-based biosensors led to improved sensitivity, lower detection limit and higher response time, better stability and rigidity, a longer shelf life, and speeding up the fabrication process [69]. There is a broad spectrum of nanomaterials, including metal nanoparticles, metal oxide nanoparticles, nanostructured polymer composites, nanofibers, nanorods, etc., utilized in the field of biosensing for polyphenol detection. Sofia et al. demonstrated numerous methods for covalent immobilization of the Lac onto several matrices (Figure 4), and concluded that the approach based on the linkage of carbon-based materials through EDC-NHS to −COOH functionalized matrices was less effective than the Lac bonded with the -NH_2_ group using glutaraldehyde to support amminated carbon based matrices [70].

## 3. Carbonaceous Nanomaterial-Based Lac Biosensor

**Figure 5:**
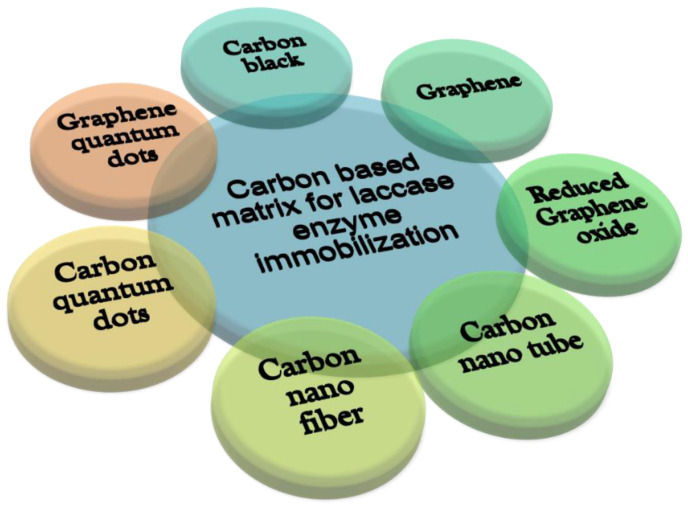
Types of carbon-based immobilization matrices.

Carbon-based nanomaterials (CNMs) are one of the most explored nanomaterials for biosensor applications. Carbon nanomaterials have comparable dimensions to redox proteins, and thus can be used as an effective electrical connector with redox enzymes. The most common CNMs used to support the Lac enzyme in biosensing applications include graphene (Gr), reduced GO, carbon nanotubes, carbon quantum dots, and graphene quantum dots (Figure 5) as summarized in Table S2. These CNMs possess a tremendous ability to detect phenolic pollutants as they have a large surface-to-volume ratio, which in addition to sensing, helps in the adsorption of pollutants. Table 2 shows the application of different CNMs-based Lac biosensors for the detection of phenols. 

### 3.1. Carbon Nano-Tube (CNT)-Based Lac Biosensors

The benzene-type hexagonal arrangement of carbon atoms forming hefty cylindrical molecules constitutes a quasi-one-dimensional CNT [110,111]. This allotrope of carbon has an enormous surface area in terms of a large length-to-diameter ratio and is observed to be just one atom thick [111]. CNTs exhibit extraordinary properties, such as being lightweight and stable, with excellent electronic, thermal, and mechanical properties [112]. A CNT lacks solubility in aqueous media, and the introduction of chemical functionalization increases their solubility in different solvents and increases their potential for biosensor applications [113]. CNTs are conjugated with many metal and metal oxide nanoparticles such as Au, Fe, Pd, Pt, Cu, Fe_3_O_4_, ZrO_2_, etc., for biosensing application [79,114]. However, certain factors such as the bundling effect, increment in the number of walls, and aggregation can cause problem while using CNTs.

### 3.2. Carbon Nanofibers (CNFs)-Based Lac Biosensors

A CNF is a one-dimensional nano range fiber with a hollow core with a diameter of 10 to 500 nm and a length in the range of 0.5 to 200 μm [115]. In CNFs, strands of layered stratified graphite sheets are stacked on a single molecule which have different variations such as platelets, ribbon, cones, herringbone, cups, etc., in the nano size range [116,117]. It has remarkable mechanical and chemical properties, due to which they are superior to other fibers. The presence of a graphitic structure enables their larger area and excellent thermal and electrical conductivities. In addition, they do not become oxidized easily. Other characteristics such as a low density, a high Young’s modulus, thermally stability, low defects, a more significant aspect ratio, and a condensed structure make CNFs suitable matrix materials [118]. These advantages lead to their applications in ceramics, fuel cells, cement composition, etc. [119]. Yang et al., used electrospinning, carbonization, and the solvothermal technique to fabricate a novel Lac-based biosensor for monitoring hydroquinone using TiO_2_ decorated copper and carbon composite nanofibers [120]. Recently, combined electrospinning and an in situ polymerization technique for synthesizing polyaniline-encapsulated carbon/copper composite nanofibers have been reported for the detection of hydroquinone in river water [87].

### 3.3. Carbon Quantum Dots (C-QDs)-Based Lac Biosensors

C-QDs are low-cost and water-soluble QDs that are easy to synthesize and have core carbon atoms organized in sp^2^ hybridization bearing oxygen-containing functional groups (hydroxyl, carboxyl, carbonyl) [121,122]. The biocompatibility, electronic properties, photostability, and photo-luminescent radiation emission of C-QDs have been explored for different applications such as photodegradation, sensing, bioimaging, drug delivery, catalysis, energy conversion, optical electronics, etc. [123].

C-QDs do not interact with all analytes directly, and very few biosensors have been reported based on C-QDs as a biosensor matrix for the detection of phenols. Recently, a fluorescent sensor has been reported utilizing the unique optical properties of C-QDs for the detection of dopamine using photo-luminescent [37]. However, the quantum yield of C-QDs is generally low, and they are difficult to reproduce due to their geometric arrangement and challenging structural characterization [124]. 

### 3.4. Graphite-Based Lac Biosensors

Graphite has been utilized as electrode material in amperometric biosensors due to its exceptional electronic and catalytic properties. Cato et al. used bare and modified graphite-epoxy decorated with either tyrosinase or the Lac enzyme and copper nanoparticles for the voltammetry detection of antioxidant activity phenols that are present in rosé cava wines [125,126]. Ibarra Escutia et al. also developed an amperometric biosensor, entrapping the fungal Lac enzyme onto polyvinyl alcohol photopolymer and graphitic SPE for sensing PhCs in tea infusions [127]. However, graphite lacs in scope for the modification of an electrode because of its lower functionality and poor solubility in organic solvents, due to which the derivatives of graphite offer more promising biosensing properties and are highly utilized.

### 3.5. Graphene (Gr) and Its Derivatives-Based Lac Biosensors

Gr is a single-layered sheet of carbon obtained after isolation from stacked layers of graphite having a thickness in atomic dimensions bearing a hexagonal layout of sp^2^ hybridized carbon atoms. Gr provides a large surface area, excellent electrical conductivity due to its highly conjugated structure, comparable tensile strength, and remarkable biocompatibility [128]. Gr-based materials provide an enhanced signal response in electrochemical biosensing [129]. Due to these properties, Gr sheets have been widely utilized as an immobilization matrix for immobilizing various enzymes, including Lac, for detecting various phenolic compounds. Palanisamy et al. fabricated a Lac enzyme-based biosensor to detect catechol using Gr cellulose microfiber composite-improved screen-printed carbon electrodes [44]. A biosensor based on the Lac enzyme immobilized on a polymer-like polyaniline (PANI) and magnetic graphene electrode has been introduced recently by Lou et al. for detecting hydroquinone [71]. 

Graphene Oxide (GO) is a derivative of Gr having abundant epoxy functional groups on the surface of Gr sheets with a large surface-to-volume ratio. Although the conductivity of GO is lower than graphene (presence of oxygen functionality), it has been reported as an immobilization matrix due to its solubility in water and its potential for the immobilization of enzymes on its surface without the use of crosslinker [130,131]. Maleki, et al. fabricated a biosensor using poly(3,4-methylenedioxy-thiophene), GO nano-sheets, and the Lac enzyme to detect catechol [75]. An electrochemical sensor utilizing GO and a molecularly imprinted polymer was developed for selective and sensitive detection of 2,4-dichlorophenol in water. It was also optimized to estimate 2,4-dichlorophenol in real water samples [132]. The introduction of oxygen functionality to GO led to a reduction in the conductivity of GO, while the debut of RGO led to better conductivity compared to that of pristine graphene. Moreover, it provides promising characteristics such as easy assembling, cost-effectiveness, higher mobility, biocompatibility, etc. [133]. A large array of materials and composites have been used for doping RGO sheets to synergistically enhance the catalytic activity and electron transfer process of biosensors for the detection of different phenolic pollutants such as 17β-estradiol [74], catechol [134], estriol [135], etc. Using RGO as a matrix for the Lac enzyme, Mei et al. fabricated RGO palladium–copper electrodes for the sensitive determination of catechol [134]. Eremia et al. developed a disposable biosensor using platinum nanoparticles/RGO composite supported on carbon screen-printed electrodes for the assessment of caffeic acid. They reported the kinetics of the detection of polyphenol electrochemically and claimed that the fabricated biosensor can be used for evaluating the total phenolic content in tea infusions [136]. Despite all the benefits, there are certain limitations of using GO and RGO such as quick agglomeration, reduction in conductivity after undergoing functionalization, and the use of toxic chemicals during synthesis. These can perhaps be overcome by the use of advanced 2D materials.

### 3.6. Graphene Quantum Dots (G-QDs)-Based Lac Biosensors

**Figure 6:**
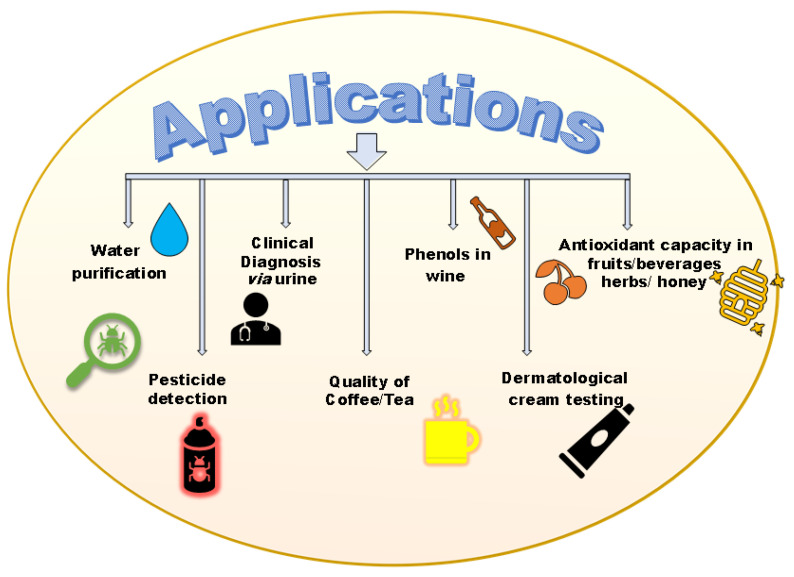
Applications of Lac enzyme-based biosensors.

G-QDs are considered zero-dimensional nanomaterials which are derived from the 2D sheet of graphene [137]. G-QDs exhibit better solubility than CNTs and displays biocompatibility, low toxicity, and stability. High quantum yield, edge effects, and confinement make it different from other carbon nanomaterials [138,139]. Top-down synthesis of G-QDs from graphene is a multistep and cost-effective process, while bottom-up synthesis of G-QDs from polycyclic aromatic compounds proceeds via complex steps and requires costly chemicals and equipment. G-QDs demonstrate remarkable photoluminescence and robust quantum properties [140]. Baluta, et al. used the fluorescence technique to detect dopamine using a Lac-based ceramic biosensor supported by graphene QDs. The fabricated biosensor was also modified using a conducting polymer [78]. Unlike graphene, G-QDs have zero band gap and can be modified using functional groups due to the edge effect [97]. Vasilescu et al. modified G-QDs electrodes using molybdenum disulfide, and the fabricated Lac-based biosensor has been used for the detection of caffeic acid and were proven to be useful for estimating the total phenolic content in red wine [141]. Inspite of several applications, the use of G-QDs is still challenging as the formation of a single layer of high-quality G-QDs is difficult. In addition, its synthesis suffers from toxic organic solvents, a long reaction time, and low yield, which limits its use for industrial purposes. Different applications of CNMs to detect PhCs have been summarized in Figure 6.

## 4. Applications of Lac-Based Biosensors

### 4.1. Detection of Phenolic Pollutants in Wastewater

Water bodies contain waste from different sources in the form of dyes and harmful chemicals that affect various life forms (both flora and fauna). Toxic chemicals, dyes, and PhCs are major raw contributors from various textile, plastics, leather, and paper industries. PhCs also merge into water bodies from the oil, gas, and coal industries. Conventional methods such as photo-catalytic degradation, distillation, ozonation, extraction techniques, membrane processes, advanced oxidation process, and enzymatic biological methods have been used to remove these dyes and phenolic pollutants from wastewater [142,143]. Before their removal, it is important to implement a real-time monitoring device to determine which pollutant is to be treated and how much to reduce the cost and time for wastewater treatment methods is very crucial. The major water pollution-contributing phenolic compounds are catechol, hydroquinone, and Bisphenol-A. 

**Figure 7:**
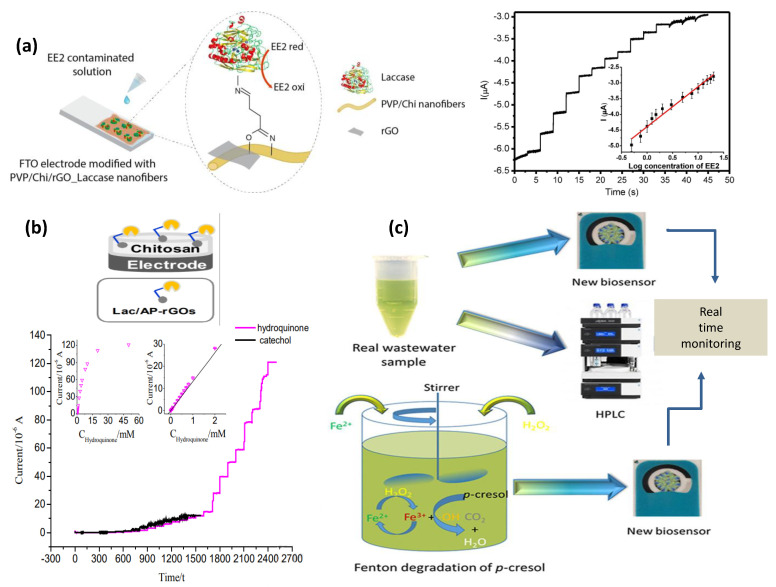
(**a**) Illustration for Lac/Polyvinylpyrrolidone/chitosan/rGO electrospun nanofibers for 17α− Ethinylestradiol electrochemical detection in synthetic and human urine. Adapted with permission from Ref. [73]. Copyright © 2018 Elsevier, (**b**) Schematic representation of Lac/aminopyrene/RGO/GCE electrode for the detection of phenols in water samples. Adapted with permission from Ref. [148]. Copyright © 2013 Elsevier (**c**) Lac/CNT/SPCE electrode for detecting p−cresol in real water samples and p−cresol produced during the Fenton process. Adapted with permission from Ref. [89]. Copyright © 2021 Elsevier.

Hydroquinone (Hq) is a widely used raw material in chemical industries as a dye intermediate and black and white photo developer and in cosmetics as a skin-lightening agent as well as a polymerization inhibitor. Studies reveal that high exposure to Hq may lead to skin-irritating effects, and it is also harmful to soil microorganisms. Recently, PANI/magnetic graphene immobilized Lac enzyme-modified electrodes were developed that show superior biosensing activity for Hq in real water samples [71]. Upan, et al. introduced a flow-injection amperometric sensor using a glutaraldehyde cross-linked Lac enzyme onto CNT-modified SPE for estimation of Hq in water sample [144]. rGO-MoS_2_ nanocomposite has been used to detect Hq using chronoamperometry technique [109]. Synthetic catechol, on the other hand, has been utilized extensively in the production of pesticides, pharmaceuticals, chemical perfumes, etc. [145,146]. Similar to Hq, it is also used in the field of black-and-white photography. Several biosensors have been developed for the evaluation of catechol. A biosensor based on an artificial neural network-integrated system has been formulated for the assessment of real water samples containing catechol under a wider linear range using poly(3,4-ethylenedioxythiophene) (PEDOT), GO nano-sheets, and Lac [75]. Kong, et al. used a graphite electrode-based intercalated montmorillonite for the electrochemical determination of catechol [147]. One of the fabricated biosensor-based on Lac/Polyvinylpyrrolidone/chitosan/rGO electrospun has been shown in Figure 7a [73]. Also, simultaneous detection of Hq and catechol has been carried out using Lac/aminopyrine/RGO/GCE electrode in water samples d [148] (Figure 7b). Bisphenol A is a component of hard plastic that are being used daily in different forms such as bottles, medical equipment, household electronic items, sports equipment, etc. [149]. Schematic representation of Lac/CNT/SPCE electrode for detecting p−cresol in real water samples and p−cresol produced during the Fenton process has been depicted in Figure 7c [89].

Bisphenol A leaches into our bodies mainly through food containers as it is used to coat the inside of food containers and drink cans. Numerous studies reveal that it acts as an endocrine disrupter even at a very low dose [150]. Bisphenol A can cause DNA damage, so monitoring its amount in water is necessary. Jalalvand, et al. used biosensing electrodes as layer-by-layer modified GCE with methylene blue-DNA/MWCNTs-chitosan/palladium nanoparticles/fullerene C60 [151]. An rGO-(4-ferrocenylethyne) phenylamine/AuNPs/GCE electrode has been used for the detection of Bisphenol A infused in milk samples [152]. Lac–thionine–carbon black-modified SPE was formulated by Portaccio, M., et al. for the assessment of endocrine disruptors in tomato juice samples [153]. Paracresol is also a water-polluting phenol, as illustrated in Figure 6a, and quite recently, a Lac/CNT/SPCE electrode has been used for its detection in real water samples. More importantly, paracresol formed while performing the Fenton process has a good resemblance with that of the results obtained using the HPLC technique [89].

One of the main causes of the accumulation of phenolic compounds in water bodies is their heavy usage in the agricultural sector as insecticides, pesticides, and herbicides. Pesticides such as pentachlorophenol undergo degradation in water into different chlorophenols. Chlorophenols easily leach from soil into water bodies as they are water-soluble and have also been widely used for impregnating wood [154]. The washing away of byproducts from agricultural materials into water bodies plays a significant role in polluting water. Biosensors help in the detection of these pesticides with ease. Carbamates are among widely used pesticides in insecticides, nematicides, fungicides, acaricides, or herbicides for increasing the yield of crops as a prophylactic measure. However, for non-targeted organisms, carbamates are toxic due to their deliberate discharge into the soil and subsequently into water bodies, leading to ill effects on the environment and human health worldwide [155]. These are also among the list of endocrine disruptors as of Bisphenol A [156], and thus become an important analyte for intensive monitoring. A novel enzyme-based biosensor has been developed by Oliveira et al. for the estimation of carbamate pesticide applied on tomato and potato crops using direct immobilization of Prussian blue functionalized Lac on graphene-modified carbon paste electrodes [157]. They also detected pirimicarb pesticide by immobilizing Lac on composite carbon paste electrodes containing an MWCNTs paste electrode modified by the dispersion of Lac within the optimum composite matrix [158]. Pesticides cause a higher threat to vegetables and fruits, which easily carry food-borne microbes and pesticide residue into our bodies when eaten raw [159]. Therefore, it is necessary to investigate the amounts of pesticides to protect the endocrine system of human beings and animals. For governing food safety, Oliveira et al. constructed a dual enzyme (Lac and tyrosinase)-based biosensor immobilized on to Au nanoparticles, chitosan, and graphene-doped carbon paste electrodes in order to detect the number of different carbamate residues in fruits [160].

### 4.2. Detection of Phenolic Compounds in Food

PhCs from natural compounds are found in vegetables, fruits, cereals, beverages, and red wines. The nutritional value of plants, fruits, and vegetables can be determined by quantifying their PhCs content [161]. It has been reported that PhCs rich food items prevent oxidative damage leading to age-associated diseases by terminating free radicals generated after cell metabolism [162]. Biosensors have proven to be an important technique to check the monitoring of food quality by detecting PhCs in plants and food products. De Macêdo, et al. estimated the total phenolic content with the help of a Lac immobilized graphite electrode in dry extracts of red fruits [163]. Honey is commonly used as an alternative to normal sugar due to its good antioxidant properties [164]. The authenticity of honey can be evaluated by inspecting its total phenolic content and antioxidant capacity using a real-time biosensor. De Oliveira Neto reported an electroanalytical method for immobilizing the Lac enzyme onto a carbon paste electrode for detecting both the antioxidant nature and total phenolic content of honey. The results correlate with the data obtained using the spectroscopic method [165]. 

Among generally consumed beverages, tea contains various PhCs (phenolic acids, flavonoids, tannins, catechins, etc.), possessing a variety of biological activities that can lead to cancer prevention, anti-aging, cardiovascular diseases, and oxidative stress in the brain. Tea infusions have excellent antioxidant properties and contain a high percentage of caffeic acid. Caffeic acid was determined from a tea infusion by Eremia, who constructed disposable biosensors based on platinum nanoparticles-RGO-Lac biocomposite electrodes [136]. Rawal et al. constructed Lac/Fe_3_O_4_NPs/cMWCNT/PANI/Au, Lac/MnO_2_NPs/cMWCNT/PANI/Au, and Lac/AgNPs/cMWCNT/PANI/Au electrodes for measuring the total content of phenols in tea leaf extract [59,166]. The total phenolic content in tea leaf extract and in black radish root was also quantified by Ibarra-Escutia, P., et al. with the help of an amperometric biosensor based on carbon SPE [167]. On the other hand, coffee, a worldwide beloved and traded beverage, holds potential for reducing high blood pressure and high cholesterol, risk to pancreatic cancer, and bone loss. All varieties of coffee contain lipids, vitamins, and carbohydrates accompanied by variable amounts of PhCs including chlorogenic acid and caffeic acid that are responsible for forecasting their virtuous qualities and categories [168]. Chlorogenic acid is the major phenol in coffee. Moreover, it is also a chief antioxidant among this beverage. GO paste/PtNPs, Lac, and BOT (botryosphaeran) electrodes were fabricated by Salamanca-Neto, et al. to discriminate the exceptional and traditional brewed coffee beverages by measuring chlorogenic acid [81].

PhCs were found to contribute to the color, astringency, mouth feel, and oxidative stabilization of wine [169,170]. PhCs in wine give an advantage to hypertensive patients based on their antioxidant nature by decreasing blood pressure [171]. Montereali et al. reported the detection of polyphenols in wine using an amperometric biosensor by immobilizing tyrosinase and the Lac enzyme onto ferrocene-modified graphite screen printed electrodes [172]. A bioelectric tongue was fabricated to detect gallic acid, a tri-hydroxy group containing compound in white wine, rose wine, and red wine [173]. The differential pulse voltametric technique has been used for assessing gallic acid using a [Cu_2_tpmc](ClO_4_)_4_/PVC matrix coated on graphite or carbon rods [174]. However, Almeida et al. [85] used thin polydopamine film on carbon electrodes for the detection of gallic acid from the chestnut shell obtained from industrial waste extract [141]. 

Phenolic content can also be tested in medicinal plants. Lac-immobilized electrodeposited MWCNT and chitosan film were used to investigate phenols (caffeic acid, gallic acids, chlorogenic acid, and rosmarinic acid) present in the medicinal plants *Salvia officinalis* and *Menthapiperita* [175]. The oil of *Menthapiperita* (Pepperment) contains specific characteristic PhCs and is a well-known medicinal ingredient that is used in tea and herbs. It has strong antioxidant properties and possesses an excellent potential as a repellent of allergies, microbes, and tumors [176]. On the other hand, the herb *Salvia officinalis* (sage) is used as tonic in medicine to improve memory, learning power, and reduce stress [177]. PhCs present in sage (Salvia officinalis) show excellent antioxidant behavior. Rosmarinic acid is the chief phenol present on both herbs [178]. Eremia et al. determined rosmarinic acid from in vitro saliva culture with Lac–Nafion-based biosensors [179]. 

### 4.3. Detection of Analytes in Body Fluids

Dopamine is a significant neurotransmitter of the brain, which plays vital roles in the cardiovascular, central nervous, and endocrine systems [180]. Dopamine is found in lower concentrations in patients with dementia, Parkinson’s disease, and schizophrenia [181]. The normal concentration of dopamine in the brain is necessary for maintaining accurate blood pressure, learning, enthusiasm, physical activities, and cognizance before releasing dopamine into the brain [37]. Therefore, a rapid clinical and sensitive diagnosis of dopamine is in considerable demand for monitoring Parkinson’s disease and depression [182]. A fluorescence-based sensor has been developed using the Lac enzyme and polydopamine on the surface of G-QDs for dopamine detection [78]. Hua, et al. assessed the role of dopamine in human urine samples using a synthesized β-Cyclodextrin inclusion complex as the immobilization matrix onto RGO for the fabrication of a biosensor [183].

Estrogens are responsible for both environmental pollution and harmful effects on humans [184]. For example, 17 β-estradiol is an environmental pollutant that is of paramount concern amongst estrogens. It is also a natural steroid that regulates the female reproductive system by contributing to estrogen [185]. Studies reveal that the release of 17β-estradiol and estriol hormones is around 280–600 μg/day and 6000–10,000 μg/day for pregnant women [156]. The presence of pollutants in food such as meat and milk lead to severe diseases related to fertility and tumor issues in females [186,187]. Biosensors for detecting estrogens from blood and urine are considered superior over other conventional methods as they are simple, rapid, and sensitive [187,188]. Wang et al. developed an electrochemical Lac enzyme poly L-lysine/citric acid-graphene biosensor to detect 17β-estradiol in urine samples [77]. Urine contains conjugated forms of estriol accompanying water soluble sulfates that hydrolyze promptly, leading to regeneration of the free form of estriol, subsequently threatening human and marine lives and giving rise to water pollution [189]. A voltammetry sensor was constructed using cobalt-poly(methionine) modified GCE for determining estriol hormone in urine and pharmaceuticals [190]. Lac functionalized polyvinylpyrrolidone/chitosan/RGO hybrid nanofibers deposited onto fluorine doped tin oxide (FTO) were electrochemically able to diagnose 17α—Ethinylestradiol in synthetic and human urine samples [73] (Figure 6c).

## 5. Future Scope

It is believed that CNTs and graphitic materials offer faster response time and appreciable sensitivity compared to traditional electrodes at minimal working potentials. However, effort has been made to modify CNMs-based biosensors to have better control on chemical and physical properties. The limitations may be overcome using the integration of metal, conducting polymers, metal chalcogenides, etc., with CNMs, which can enhance the performance of biosensors to address future challenges. 

A lot of interest is now been focused on advanced 2D materials such as MXenes (MXe), which was recently explored in the field of biosensors [191]. MXe has been found to be better than graphene-based nanomaterials in terms of improved electron transfer in the heterogeneous phase, low diffusion barrier, and stable dispersibility. This is due to the presence of metal at the center on MX, which does not reduce its conductivity or dispersibility even after functionalization and offers excellent biocompatibility. In this context, MXe based biosensors are progressing toward the development of lab-on-chip biosensing devices involving 5G communication [192]. 

Future generations demand quick and accurate detection of analytes [193]. New aspects such as the internet of things and machine learning involving artificial intelligence (AI) are being used in the advancement of point-of-care (POC) devices which are emerging more promising technologies as compared to conventional healthcare monitoring [194]. Emphasis on advanced biosensor fabrication must be given to use AI for detecting phenols and for their on-site and online monitoring in order to balance the content of phenol in diverse applications, as discussed in this review article.

## 6. Conclusions

The reliable detection of PhCs using Lac-based biosensors may result in maintaining permissible polyphenols in water, body fluids, and sustainable management of antioxidant quality. Developing a biosensor for PhCs detection will provide a way to overcome conventional techniques, which are time-consuming and expensive. The application of Lac-based biosensors is mainly focused on the electrochemical transduction method. Amperometric biosensors are among the most reported techniques for detecting PhCs using the Lac enzyme and carbonaceous matrices. The carbon-based matrix has shown enormous potential to support the Lac enzyme by providing enhanced surface area, superior conductivity, stability, and excellent mechanical strength. They have been used in diverse applications to detect polyphenols from wastewater, wine samples, urine, fruits, vegetables, juices, human serums, and beverages. The most significant limitation that remains is the leaching of the enzyme from the substrate. This can perhaps be improved using suitable immobilization techniques and incorporation of nanomaterials to maintain the enzyme activity. The integration of CNMs with Lac-based biosensors can be crucial in developing POC devices for application in food, pharmaceuticals, and environmental monitoring. The oxidizing characteristics of Lac make it an effective biocatalyst, which can also be utilized for other applications related to the same mechanism. Enzyme modification, the multienzyme approach, AI, and advanced 2D materials should be explored for the development of POC devices.

## Figures and Tables

**Table 2:** Biosensors used for the detection of phenols using the Lac enzyme.

S.No.	Matrix	Method of Analysis	Analyte	Matrices	Linear Range	LOD	Ref.
1.	PANI/MG composite	ChronoAmp	Hydroquinone	Gr	0.4–337.2 μM	2.94 μM	[71]
2.	Microporous carbon fibers	ChronoAmp	Catechol	C F	0.01–0.05 mM	<5 μM	[36]
3.	Au NP/graphene NP-SPE	ChronoAmp	Hydroquinone	Gr	4–130 µM	1.5 µM	[72]
4.	Polyvinylpyrrolidone/CS/RGO	Amp	17 α-Ethinylestradiol	RGO	0.25–20 pmol L^−1^	0.15 pM	[73]
5.	Rh/GO	DPV	17β-estradiol	GO	0.9–11 pM	0.54 pM	[74]
6.	PEDOT, GO nano-sheets	DPV	Catechol	GO	0.036–0.35 μM and 0.35–2.5 μM,	0.032 μM	[75]
7.	RGO-MWCNT	ChronoAmp	Epicatechin equivalents	RGO & MWCNT	1–300 μM	0.3 μM	[76]
8.	Poly L-lysine/Citric acid-Gr/GCE	DPV	17β-estradiol	Gr	4 × 10^−13^–5.7 × 10^−11^ M	1.3 × 10^−13^ M	[77]
9.	Poly(dithienotetraphenylsilane)	Fluorescence	Dopamine	G-QDs	1–200 μM	80 nM	[78]
10.	SPCE/anthraquinone-COOH-MWCNT	Amp	Catechol	MWCNT	0.002–0.061 μM		[79]
11.	G/PANABA/MWCNT	ChronoAmp	Phenol	G & MWCNT	0.0005–0.4 mM	0.5 μM	[65]
12.	Bacterial Cellulose/cMWCNTs/ZIF-8		Bisphenol A	MWCNT	0.01–0.4 mM	1.95 mM	[80]
13.	Pt NP/GO	SWV	Chlorogenic acid	GO	0.56–7.3 µmol L^−1^	0.18 and 0.59 µM	[81]
14.	Au–ZnO/NP/ITO	Amp	Catechol	NC	75 nM–1100 μM	25 nM	[82]
15.	D glucan/carbon black paste/Au NP	SWV	Hydroquinone	C	2.00–56.5 μM	0.474 μM	[83]
16.	F, N-doped carbon dots	CV	Catechol	F,N-CD	0.1–0.45 mM	0.014 μM	[84]
17.	Thin polydopamine film/carbon surfaces	ChronoAmp	Caffeic acid, rosmarinic acid, and gallic acid	C	1–150 μM	0.29 μM	[85]
18.	PPy/GCE	Amp	Catechol	GCE	1–60 μM		[86]
19.	PANI/ CuCNFs	Amp	Hydroquinone	C/Cu NF	500 nM–110 μM	0.24 μM	[87]
20.	BMIMBF4-CS and MWCNT	SWV	Bisphenol A	MWCNT		8.4 ± 0.3 nM	[88]
21.	Plastic packaging waste derived CNTs/SPCE		Para cresol	CNT	0.2–25 ppm	0.05 ppm	[89]
22.	Au-MXene (Ti_3_C_2_)	Amp	Catechol	Ti_3_C_2_	0.05–0.15 µM	0.05 µM	[90]
23.	Enzyme POXA1b and POXA1b-Vmh2/MWCNT	ChronoAmp	Catechol and Dopamine	MWCNT	2–30 pM, 0.1–800 μM and 0.015–90 μM	2 pM and 15 nM	[91]
24.	BC/c-MWCNTs/LAC@ZIF-90 membrane	DPV	Catechol	MWCNT	20–400 μM	1.86 µM	[92]
25.	CMB/CBPE	SWV	Quercetin	Carbon black	4.98–50.0 × 10^−8^ M	2.6 × 10^−8^ M	[93]
26.	OMC-SPE	SWV	Serotinin	Mesoporous carbon	0.1–1.2 mu M	316 nM	[94]
27.	TiO_2_/nafion/graphitic	CV	Gallic acid	G	0.125–175 mu M	0.125 mu M	[95]
28.	Fe_3_O_4_-Pc-cMWCNTs	DPV	Rosenmerinic acid	cMWCNT	0.2–400 mu M	0.182 mu M	[96]
29.	MnO_2_/GNP decorated SPCE	Amp	Caffeic acid	GNP	0.3 µM–0.4 mM	1.9 mu M	[97]
30.	Cellulase/c-MWCNTs	Amp	Catechol	cMWCNT	10–160 mu M	0.004 mu M	[98]
31.	Au-RGO/SPE	DPV	Catechol	RGO	1 mM–1 nM	3.3 µM	[99]
32.	SPCE modified Gr-AuNPs with CS	DPV	Bisphenol A	Gr	0.05–12 µM	0.023 mu M	[100]
33.	Ag-ZnO/ MWCNTs/SPE	DPV	Bisphenol A	MWCNT	0.5–2.99 mu M	6 nM	[101]
34.	CS-Fe_2_O_3_/RGO	ChronoAmp	Bisphenol A	RGO	6–228 ppb	18 nM	[102]
35.	GO/Fe Pc composite		Adrenaline	GO	1.8–92 µM		[103]
36.	TiO_2_-GPE	DPV	Methyldopa	GPE	10–180 mu M	1 mu M	[104]
37.	G-QDs		Catecholamine	G-QDs	1–120 µM	83 nM	[105]
38.	Gr/Cu/Fe_3_O_4_ composites	DPV	Bisphenol A	Gr	7.2–18 µM	1.7 µM	[106]
39.	PEDOT/Au/cMWCNT		Catechol	cMWCNT	0.1–0.5 and 11.99–94.11 µM	0.11 and 12.26 µM	[107]
40.	Gr/PPy nanotubes/SrCuO_2_	DPV	2,4-Di chlorophenol	Gr	1–50 mu M	0.18 mu M	[108]
41.	RGO-MoS_2_	ChronoAmp	Hydroquinone	RGO	1–100 µM	0.1 µM	[109]

## Data Availability

Data will be made available on request.

## Supplementary Materials

The following supporting information can be downloaded at: https://www.mdpi.com/article/10.3390/bios13030305/s1, Table S1: Sources of the laccase enzyme; Table S2: Doping materials used in carbon matrices [195,196,197,198,199].

## Abbreviations

PANI: Polyaniline, NP: nanoparticle, SPE: screen printed electrode, SPCE: screen printed carbon electrode, TPC: total phenolic content, GCE: glassy carbon electrode, CS: chitosan, ZIF: zeoliticimid–azolate framework, BC: bacterial cellulose, Pc: phthalocyanine, CMB: carboxymethyl-botryosphaeran, CBPE: carbon black paste electrode, GNPl: graphenenanoplatelets, BMIMBF4: 1-butyl-3-methylimidazolium tetra-fluoroborate, PVP: polyvinylpyrrolidone, NF: nanofiber, GPE: graphene doped carbon paste electrode, Tyr: Tyrosinase, G: graphite, PANABA: poly-aniline-2-aminobenzylamine, OMC-SPE: organized mesoporous carbon-modified carbon screen-printed electrode, cMWCNT: carbonylated multi wall carbon nanotube, MG: magnetic graphene, PEDOT: poly(3,4-ethylenedioxythiophene), PPy: polypyrrole, G-QDs: graphene quantum dots, POXA1b: laccase Pleurotus ostreatus, DPV: Differential Pulse Voltametry, CV: Cyclic voltammetry, SWV: square wave voltammetry, Amp: Amperometry, ChronoAmp: Chronoamperometry
