# Supplementary material for: Recent Prospects of Carbonaceous Nanomaterials-Based Laccase Biosensor for Electrochemical Detection of Phenolic Compounds
Source: Biosensors (Basel). 2023 Feb 22;13(3):305. doi: 10.3390/bios13030305 (PMC10046707; doi:10.3390/bios13030305)
Supplement: Supplementary file 1 [file biosensors-13-00305-s001.zip › biosensors-2191704-supplementary.pdf]

Recent Prospects of Carbonaceous Nanomaterials-Based Laccase Biosensor for Electrochemical Detection of Phenolic Compounds

Sakshi Verma [1], Deeksha Thakur [1], Chandra Mouli Pandey [2,*] and Devendra Kumar [1,*]

[1] Department of Applied Chemistry, Delhi Technological University, Delhi 110042, India
[2] Department of Chemistry, Faculty of Science, SGT University, Gurugram 122505, India
* Correspondence: cmp.npl@gmail.com (C.M.P.); dkumar@dce.ac.in (D.K.)

Table S1. Sources of Laccase enzyme.

Plants<br>• Fruits such as apples, peach, pears etc.<br>• Vegetables such as cabbage, potatoes, beets.<br>• Other plants like tobacco, sycamore, maize's embryo and many more [1]	Fungi<br>• Ascomycetes (Polyporousver-cicolor, Aspergillusnidulan),<br>• deuteromycetes (Pestalotiopsis species)<br>• basidiomycetes (white rot fungi, Tremetes fungi etc) are major sources. [2]·[3]
Insects<br>• Azospirillumlipoferum (Ist reported)<br>• Other insects such as Bacillus subtilis (Gangola, Sharma, Bhatt, Khati, & Chaudhary,2018),Streptomyces cyaneus [4], Streptomycesipomoeae[5], Streptomyces lav-endulae [6], Streptomyces coelicolor [7], and Thermusthermophilus[8]	Bacteria<br>• In particular Azospiril-lumlipoferum, Pseudomonas putida F6,Bacillus licheniformis, Bacillus subtilis WPI [9]

Table S2. Doping materials used in carbon matrices.

S.No.	Matrix material	Doping materials	References
1.	Carbon black	PANI, Thionine	[10]
2.	Carbon quantum dots	F, N	[11]
3.	Graphite	Epoxy, PVA, Ferrocene	[12-14]
4.	Graphene	PANI, Au, Pt	[15-17]
5.	Graphene oxide	Rh, PEDOT	[18, 19]
6.	Reduced graphene oxide	Pd/Cu, Sb_2O_5, Pt, PVP/CS	[15, 20-22]
7.	Carbon nano fibers	TiO_2	[23]
8.	Carbon nano tubes	Cellulose, CS, Ni/PANI, MnO_2, Au	[24-28]
9.	Graphene quantum dots	MoS_2	[29]

References

1. Kumar, V. and P. Sonkar, *LACCASES: SOURCES AND THEIR ENVIRONMENTAL APPLICATION.* International Journal of Bioassays, 2013. **2**: p. 909-911.
2. Viswanath, B., et al., *Fungal Laccases and Their Applications in Bioremediation.* Enzyme Research, 2014. **2014**: p. 163242.
3. Shraddha, et al., *Laccase: Microbial Sources, Production, Purification, and Potential Biotechnological Applications.* Enzyme Research, 2011. **2011**: p. 217861.

4. Ece, S., et al., *Heterologous expression of a Streptomyces cyaneus laccase for biomass modification applications.* AMB Express, 2017. **7**(1): p. 86-86.
5. Blánquez, A., et al., *Laccase SilA from Streptomyces ipomoeae CECT 3341, a key enzyme for the degradation of lignin from agricultural residues?* PloS one, 2017. **12**(11): p. e0187649-e0187649.
6. Mishra, S. and D.S. Srivastava, *Production and Optimization of Laccase from Streptomyces lavendulae.* International Journal of Current Microbiology and Applied Sciences (IJCMAS), 2017. **6**: p. 1239-1246.
7. Majumdar, S., et al., *Roles of Small Laccases from Streptomyces in Lignin Degradation.* Biochemistry, 2014. **53**(24): p. 4047-4058.
8. Miyazaki, K., *A hyperthermophilic laccase from Thermus thermophilus HB27.* Extremophiles, 2005. **9**(6): p. 415-425.
9. Chauhan, P.S., B. Goradia, and A. Saxena, *Bacterial laccase: recent update on production, properties and industrial applications.* 3 Biotech, 2017. **7**(5): p. 323-323.
10. !!! INVALID CITATION !!! .
11. Liu, L., et al., *Electrochemical sensor based on F,N-doped carbon dots decorated laccase for detection of catechol.* Journal of Electroanalytical Chemistry, 2019. **840**: p. 84-92.
12. Cetó, X., F. Céspedes, and M. del Valle, *BioElectronic Tongue for the quantification of total polyphenol content in wine.* Talanta, 2012. **99**: p. 544-551.
13. Ibarra-Escutia, P., et al., *Amperometric biosensor based on a high resolution photopolymer deposited onto a screen-printed electrode for phenolic compounds monitoring in tea infusions.* Talanta, 2010. **81**(4): p. 1636-1642.
14. Montereali, M.R., et al., *A disposable Laccase–Tyrosinase based biosensor for amperometric detection of phenolic compounds in must and wine.* Journal of Molecular Catalysis B: Enzymatic, 2010. **64**(3): p. 189-194.
15. Eremia, S.A.V., et al., *Disposable biosensor based on platinum nanoparticles-reduced graphene oxide-laccase biocomposite for the determination of total polyphenolic content.* Talanta, 2013. **110**: p. 164-170.
16. Lou, C., et al., *Laccase immobilized polyaniline/magnetic graphene composite electrode for detecting hydroquinone.* Int J Biol Macromol, 2020. **149**: p. 1130-1138.
17. Zrinski, I., et al., *Evaluation of phenolic antioxidant capacity in beverages based on laccase immobilized on screen-printed carbon electrode modified with graphene nanoplatelets and gold nanoparticles.* Microchemical Journal, 2020. **152**: p. 104282.
18. Maleki, N., et al., *A Novel Enzyme Based Biosensor for Catechol Detection in Water Samples Using Artificial Neural Network.* Biochemical Engineering Journal, 2017. **128**.
19. Povedano, E., et al., *Decoration of reduced graphene oxide with rhodium nanoparticles for the design of a sensitive electrochemical enzyme biosensor for 17β-estradiol.* Biosensors and Bioelectronics, 2017. **89**: p. 343-351.
20. Cincotto, F.H., et al., *Reduced graphene oxide-Sb2O5 hybrid nanomaterial for the design of a laccase-based amperometric biosensor for estriol.* Electrochimica Acta, 2015. **174**: p. 332-339.
21. Mei, L.-P., et al., *Novel phenol biosensor based on laccase immobilized on reduced graphene oxide supported palladium–copper alloyed nanocages.* Biosensors and Bioelectronics, 2015. **74**: p. 347-352.
22. Pavinatto, A., et al., *Ultrasensitive biosensor based on polyvinylpyrrolidone/chitosan/reduced graphene oxide electrospun nanofibers for 17α – Ethinylestradiol electrochemical detection.* Applied Surface Science, 2018. **458**: p. 431-437.
23. Yang, J., et al., *TiO2-CuCNFs based laccase biosensor for enhanced electrocatalysis in hydroquinone detection.* Journal of Electroanalytical Chemistry, 2016. **766**: p. 16-23.
24. Chawla, S., et al., *An amperometric biosensor based on laccase immobilized onto nickel nanoparticles/carboxylated multiwalled carbon nanotubes/polyaniline modified gold electrode for determination of phenolic content in fruit juices.* Biochemical Engineering Journal, 2012. **68**: p. 76-84.
25. Diaconu, M., S.C. Litescu, and G.L. Radu, *Laccase–MWCNT–chitosan biosensor—A new tool for total polyphenolic content evaluation from in vitro cultivated plants.* Sensors and Actuators B: Chemical, 2010. **145**(2): p. 800-806.
26. El Ichi-Ribault, S., et al., *Performance and stability of chitosan-MWCNTs-laccase biocathode: Effect of MWCNTs surface charges and ionic strength.* Journal of Electroanalytical Chemistry, 2017. **799**: p. 26-33.
27. Li, X., et al., *Encapsulation of enzyme by metal-organic framework for single-enzymatic biofuel cell-based self-powered biosensor.* Nano Energy, 2020. **68**: p. 104308.
28. Rawal, R., et al., *An amperometric biosensor based on laccase immobilized onto MnO2NPs/cMWCNT/PANI modified Au electrode.* International Journal of Biological Macromolecules, 2012. **51**(1): p. 175-181.
29. Vasilescu, I., et al., *Molybdenum disulphide and graphene quantum dots as electrode modifiers for laccase biosensor.* Biosensors and Bioelectronics, 2016. **75**: p. 232-237.
